# Investigating group-based classes (‘weaning workshops’) to support complementary infant feeding in Irish primary care settings: a cross-sectional survey

**DOI:** 10.1017/S1368980022000477

**Published:** 2022-08

**Authors:** Caragh Flannery, Caroline Shea, Yvonne O’Brien, Joanne O’Halloran, Karen Matvienko-Sikar, Colette Kelly, Elaine Toomey

**Affiliations:** 1 School of Public Health, University College Cork, Cork T12 K8AF, Ireland; 2 INFANT Centre, Cork University Maternity Hospital, University College Cork, Cork, Ireland; 3 Community Nutrition and Dietetic Service, Cork Kerry Community Healthcare, HSE, Cork, Ireland; 4 Primary Care Centre, Mountkennedy Town Centre, Newtownmountkennedy, Co Wicklow, Ireland; 5 Health Promotion Research Centre, National University of Ireland Galway, Galway, Ireland; 6 School of Allied Health, University of Limerick, Co. Limerick, Ireland; 7 Health Research Institute, University of Limerick, Co. Limerick, Ireland

**Keywords:** Weaning workshops, Complementary infant feeding, Infant feeding, Primary care

## Abstract

**Objective:**

This study aims to (1) investigate current practice regarding ‘weaning workshops’ to support complementary infant feeding delivered within Irish primary care, (2) explore the experiences and opinions of community dietitians regarding optimal content and modes of delivery of weaning workshops and (3) identify the key factors to be considered in the development and implementation of weaning workshops delivered within primary care.

**Design:**

Cross-sectional survey.

**Setting:**

Irish primary care.

**Participants:**

Forty-seven community-based dietitians.

**Results:**

Sixteen dietitians reported that workshops were run in their area with variable frequency, with ten reporting that workshops were never run in their area. Participants reported that mostly mothers of medium socio-economic status (SES) attended weaning workshops when infants were aged between 4 and 7 months, and that feedback from workshop attendees was predominantly positive. Dietitians identified that key factors to be considered in future development and delivery of weaning workshops are (1) workshop characteristics such as content, timing and venue, (2) organisational characteristics such as availability of resources and multidisciplinary involvement and (3) attendee characteristics such as SES.

**Conclusions:**

This study highlights substantial variability regarding provision of weaning workshops in Ireland, and a lack of standardisation regarding the provider, content and frequency of workshops where workshops are being delivered. The study also provides unique insights into the experiences and opinions of primary care community dietitians regarding the development and delivery of weaning workshops in terms of optimal content and delivery options. These perspectives will make a valuable contribution given the dearth of evidence in this area internationally.

Optimum infant nutrition is a vital aspect of healthy child development and chronic disease prevention, and the complementary introduction of solid foods within the first 2 years of life plays a key role in this. International WHO infant feeding guidelines recommend that infants be exclusively breastfed until 6 months, with nutritionally adequate and appropriate solid foods introduced from 6 months onwards^(1,2)^. However, despite these recommendations, a substantial proportion of infants globally are introduced to solids before 4 months^(3)^. In Ireland, a recent study identified that 18 % of Irish infants had begun complementary feeding before 17 weeks, with another showing 13·5 % taking solids between 12 and 16 weeks^(4–6)^. Previous research also found that a high proportion of Irish infants aged 6 months consume foods high in energy, salt, saturated fats and refined sugars^(7)^. Given the importance of establishing healthy dietary behaviours from childhood, supporting parents and primary caregivers to introduce solid foods for infants and young children in line with best practice recommendations is of key significance both nationally and internationally.

Multiple factors influence complementary infant feeding behaviours. For example, Fall *et al.*^(8)^ identified several factors associated with the early introduction of solids, including younger maternal age, lower socio-economic status (SES), lower maternal education, absent/short duration of breastfeeding and maternal smoking. More recently, Gutierrez-Camacho *et al.*^(9)^ identified that higher maternal education, higher age and higher household income are positively associated with healthy infant feeding behaviours. In addition, recent qualitative evidence syntheses explored parents’ perceptions and experiences of infant feeding and identified many factors influencing parents’ complementary feeding behaviours^(10,11)^. Both reviews found that complementary feeding behaviours are influenced by multiple sources of advice, in particular those of peers (i.e. family and friends) and healthcare professionals. The studies identified a need for greater education and support for parents regarding complementary feeding, particularly within primary care settings^(11)^, and considering practical issues such as time and cost^(10)^. The reviews also highlighted greater barriers within lower SES populations, such as the lack of affordability of healthy food^(12)^, greater misconceptions and lack of knowledge^(10)^.

Despite an increasing focus on the importance of appropriate complementary infant feeding, and the need to support parents and primary caregivers to engage in recommended behaviours, the existing evidence regarding the effectiveness of interventions to improve complementary feeding behaviours is inconsistent^(10–12)^. Recent systematic reviews of infant feeding interventions have shown minimal effects on infant feeding practices and other important outcomes such as child weight^(10,12,13)^. Methodological limitations of existing interventions have also been highlighted with poor application and/or use of behaviour change theory, a lack of systematic intervention development and a lack of focus on long-term implementation and sustainability^(13–15)^. Results of these reviews have emphasised the importance of trustworthy, non-biased, consistent infant feeding information for parents while also facilitating the development of practical skills and providing hands-on support such as demonstrating how to prepare nutritional meals^(10,12–15)^.

In Ireland, programmes such as the National Healthy Childhood Programme (NHCP) and the Nurture Programme aim to provide guidance, advice and support for families during early childhood on topics including infant feeding^(16,17)^. Furthermore, an important focus of these programmes is the use of existing healthcare contact points to provide support and advice and improve consistency in information delivery by healthcare professionals. Given the important role that healthcare professionals can play in influencing infant feeding behaviours coupled with the strong social influence of family and friends^(3,12,13)^, the current evidence therefore suggests a need for rigorously developed and evaluated sustainable community-based interventions that can be embedded into routine healthcare services. Primary care represents a promising setting for such interventions given its community-based context and an increasing global focus on health promotion and disease prevention within primary care practice^(18–20)^. Given their expertise, community dietitians may have an important role to play in interventions that aim to improve healthy infant feeding; however, recent research has highlighted limited capacity amongst dietitians to prioritise preventive-focused interventions over complex clinical issues such as feeding challenges or problems^(21)^.

Group-based parenting programmes have been previously recognised as an effective means to change parents’ behaviour, and when implemented in community-based settings, have been found to improve child social, emotional outcomes, as well as parenting skills^(22–24)^. Furthermore, these group-based programmes have shown that they can be delivered with high levels of implementation fidelity and parental engagement^(22,25,26)^. Other studies have identified factors which are associated with the success of parenting programmes including parental attitudes towards programme content, changes in parenting skills and confidence and positive experiences of the group process^(26–28)^. In the context of early child nutrition, group-based programmes focusing on complementary feeding, or ‘weaning workshops’, have been suggested as a promising approach to target infant feeding practices^(10,21,23,24,29,30)^. Such programmes focus on individual- or family-level behaviour change through a number of home visits, individual counselling or group sessions in a clinical or community setting targeting several important aspects e.g. access to evidence-based information, social support and practical skills^(31)^. Furthermore, these interventions have demonstrated improvements in breast-feeding practices, and in specific complementary feeding practices such as improved dietary diversity and age at introduction of complementary foods^(10,14,28)^. However, apart from some notable exceptions^(29,30)^ there is limited research on the development, implementation and evaluation of weaning workshops within community or primary care settings, with the majority of evidence focusing predominantly on breastfeeding support or individually delivered interventions^(32,33)^. Additionally, current practice surrounding weaning workshops, optimum content, effective mode(s) of delivery timing and frequency of the interventions have been poorly explored to date, both in Ireland and internationally.

In Ireland, infant feeding education is provided in community settings by public health nurses and dietitians with little known about the underlying aims of these workshops/classes or how they are developed and implemented. Furthermore, private infant feeding education is also available, but these classes are not always provided by healthcare professionals. A previous unpublished report from the Irish Health Service Executive in 2016 completed by eleven dietitians from nine Irish community health organisation (CHO) areas identified that weaning workshops were being offered in 7/9 CHO areas, and that programme content and implementation were highly variable across settings (Health Service Executive Weaning Workshop, unpublished data). Ten of the included dietitians highlighted the need for standardised weaning workshop; however, this report did not collect information from participants regarding their views on the optimum content or mode(s) of delivery of weaning workshops, nor the factors that need to be considered in their development and implementation in primary care settings. Due to the potential for community-based weaning workshops to support complementary infant feeding behaviours and the scant existing evidence internationally to inform appropriate development and implementation, further exploration of such interventions is needed.

This study aims to:

Investigate current practice regarding the delivery of weaning workshops within Irish primary care settings,

To explore the experiences and opinions of community dietitians regarding the optimal content and modes of delivery of weaning workshops and

To identify the key factors that should be considered to facilitate the development and implementation of effective and sustainable weaning workshops within routine primary care from the perspectives of community dietitians.

## Methods

### Study design

The research adopted a quantitative approach using a cross-sectional survey distributed to community-based dietitians working in the Irish primary healthcare service. We obtained ethical approval from the School of Public Health Internal Social Research Ethics Committee in University College Cork. The STROBE checklist for cross-sectional studies^(34)^ was used to inform reporting of the findings (Appendix 1).

### Sampling and recruitment

All community-based Health Service Executive dietitians based in Ireland were eligible to participate in the study. After consultation with a senior community dietitian in primary care (J.O.H.), it was established that there are approximately ninety community-based dietitians in Ireland were eligible to participate in the study. The survey was set up using ‘Lime Survey’, an online resource for hosting surveys and questionnaires. An email inviting all community-based Health Service Executive dietitians in Ireland was sent by a primary care dietitian manager (Y.O.B.) in the Cork area from May to June 2019. One reminder email was sent within this timeframe. The invitation email included an information sheet outlining the nature of the research, a link to the survey and instructions for obtaining consent. Before emailing, the survey was piloted with two participants (a researcher with a nutritional background and a dietitian), after which minor refinements were made to the wording of questions. The pilot responses were not included in the analysis.

### Data collection

A thirty-four-item survey was developed based on existing literature in conjunction with an unpublished report by the Health Service Executive^(10,29,35)^. In the first section, participants were asked to provide their age category, gender, CHO area, length of time qualified, whether they worked in primary care or health promotion and current position. The second section asked participants about current weaning workshops service offerings in their CHO area. Specifically, participants were asked questions about workshop characteristics including frequency, provider, content and resources required and about attendee characteristics, e.g. numbers in attendance, average parent/caregiver type (e.g. mothers, fathers, grandparents etc), average SES, average age of infant and their views on the general reception by attendees regarding the workshops. The next section asked participants about their specific experience and opinions of delivering weaning workshops, and their views of the factors influencing the development and delivery of weaning workshops and attendance by parents/caregivers. All of the above items were closed-ended questions with multiple-choice answers provided (*n* 30 items). Four open-ended questions were also included to capture participants’ views on (1) reasons that workshops are currently not available in their area (if applicable), (2) the most important factors to consider in development and delivery of weaning workshops, (3) recommendations regarding existing services and (4) any additional comments. We defined weaning workshops for participants as ‘any community-based, group-based classes that aim to address complementary feeding and the introduction of solid foods for parents and primary caregivers of infants and children’. The full questionnaire is provided in Appendix 2.

### Data analysis

The data were cleaned and then transferred into STATA (version 13) for analysis. A descriptive univariate analysis was conducted by C.F. for all dietitians’ characteristics, workshop characteristics and reported attendee characteristics, providing sample numbers and percentages. We used chi-squared test for categorical variables to present differences between responses from dietitians who had the experience of delivering workshops and those who did not, but we did not calculate significance or p-values due to the small samples involved. All surveys were returned complete but scattered missing data reduced the sample size for some variables (further data provided on missing data in Appendix 3). In items that allowed for multiple responses (‘tick all that apply’), categories were created for most of the matching responses. For all additional responses, an ‘other’ category was created. Data from all four open-ended questions were analysed by E.T. using a conventional content analysis approach outlined by Hsieh and Shannon^(36)^. This approach was chosen due to the likelihood of minimal depth and richness of responses. After reading and data familiarisation, E.T. highlighted text that captured key concepts and meaning from responses. These key concepts were then summarised, producing initial codes, which were refined and sorted into overarching categories which were subsequently discussed with C.F.

## Results

### Dietitians’ characteristics

A total of ninety dietitians were invited to participate in this study; 47 (52 %) consented to participate. Most participants were in the 31–40 years of age bracket (*n* 17, 39·5 %). Community dietitians were predominantly female (*n* 41, 97·6 %), based in primary care (*n* 33, 80·5 %) and working as a dietitian for 1–5 years (*n* 14, 35 %). Representation varied across the nine CHO areas represented, ranging from 0 (CHO area 2) to 12 (28·6 %) from CHO area 4. Most participants were in a senior dietitian role (*n* 31, 73·8 %) (See Table 1).

**Table 1:** Dietitians’ characteristics

Variable	*n*	%
Age category (*n* 43)		
18–30 years	7	16·3
31–40 years	17	39·5
41–50 years	14	32·6
51–60 years	4	9·3
> 60 years	1	2·3
Gender (*n* 42)		
Female	41	97·6
Male	1	2·4
CHO (*n* 42)		
Area 1	3	7·1
Area 2	0	0·0
Area 3	3	7·1
Area 4	12	28·6
Area 5	4	9·5
Area 6	7	16·7
Area 7	5	11·9
Area 8	1	2·4
Area 9	7	16·7
Length in profession (*n* 40)		
1–5 years	14	35·0
6–10 years	5	12·5
11–15 years	10	25·0
16–20 years	10	25·0
21–25 years	1	2·5
Discipline (*n* 41)		
Primary care	33	80·5
Health promotion	4	9·8
Other	4	9·8
Current position (*n* 42)		
Dietitian manager	3	7·1
Senior dietitian	31	73·81
Staff-grade dietitian	8	19·1

CHO, Community Health Organisation; Area 1, Donegal, Sligo/Leitrim/West Cavan, Cavan/Monaghan LHO; Area 2, Community Healthcare West; Area 3, Mid-West Community Healthcare; Area 4, Cork Kerry Community Healthcare; Area 5, South East Community Healthcare; Area 6, Community Healthcare East; Area 7, Community Healthcare Dublin South, Kildare & West Wicklow; Area 8, Midlands Louth Meath Healthcare; Area 9, Dublin North City and County Healthcare.

### Dietitians’ characteristics and experience of delivering weaning workshops

Data for delivery of weaning workshops were available for thirty participants, with most dietitians reporting not having delivered weaning workshops (*n* 19, 63·3 %) (Table 2). Of the dieticians that had personally delivered weaning workshops previously, most were aged between 41 and 50 years (*n* 6, 54·6 %), based in primary care (*n* 9, 81·8 %) and in a senior dietitian’s role (*n* 9, 81·8 %). Characteristics were mostly similar for dietitians who had not delivered workshops previously, with the majority aged between 31 and 40 years (*n* 8, 42·1 %), based in primary care (*n* 14, 73·7 %) and in a senior dietitian role (*n* 14, 73·7 %). The findings of chi-squared tests are presented in Tables 2–4, with data for dieticians with experience of delivery presented separately to those without experience.

**Table 2:** Dietitians’ characteristics and delivery of weaning workshops (WW)

		Delivered a WW (*n* 30)			
		Yes (*n* 11, 36·7 %)		No (*n* 19, 63·3 %)	
	*n*	*n*	**%**	*n*	**%**
Age category	30				
18–30 years		1	9·1	3	15·80
31–40 years		4	36·4	8	42·1
41–50 years		6	54·6	6	31·6
51–60 years		0	0·0	1	5·3
> 60 years		0	0·0	1	5·3
Gender	30				
Female		11	100·0	18	94·7
Male		0	0·0	1	5·3
CHO	30				
Area 1		2	18·2	1	5·3
Area 2		0	0·0	0	0·0
Area 3		0	0·0	3	15·8
Area 4		2	18·2	4	21·1
Area 5		0	0·0	4	21·1
Area 6		2	18·2	4	21·1
Area 7		2	18·2	1	5·3
Area 8		0	0·0	0	0·0
Area 9		3	27·3	2	10·5
Length of time in profession	30				
1–5 years		2	18·2	9	47·4
6–10 years		2	18·2	2	10·5
11–15 years		3	27·3	4	21·1
16–20 years		4	36·4	4	21·1
21–25 years		0	0·0	0	0·0
Discipline	30				
Primary care		9	81·8	14	73·7
Health promotion		2	18·2	2	10·5
Other		0	0·0	3	15·8
Current position	30				
Dietitian manager		1	9·1	1	5·3
Senior dietitian		9	81·8	14	73·7
Staff-grade dietitian		1	9·1	4	21·1
Parents looking for support	27				
Daily		2	18·2	0	0·0
Once a week		4	36·4	4	25·0
Once a month		3	27·3	6	37·5
Few times a year		2	18·2	2	12·5
Never		0	0·0	4	25·0

CHO, Community Health Organisation.

**Table 3:** Dietitians’ perceptions of those attending weaning workshops (WW) and delivery of WW

		Delivered a WW (*n* 30)			
		Yes (*n* 11, 36·7 %)		No (*n* 19, 63·3 %)	
Variable	*n*	*n*	**%**	*n*	**%**
How many attend WW (*n* 26)	22				
≤ 10 participants		5	45·5	4	36·4
> 10 participants		6	54·6	2	18·2
Do not know		0	0·0	5	45·5
Who attends WW (*n* 28)	24				
Both mother and father		2	18·2	0	0·0
Mother only		9	81·8	6	46·2
Do not know		0	0·0	7	53·9
Parents SES (*n* 26)	23				
High		0	0·0	2	16·7
Medium		9	81·8	4	33·3
Low		0	0·0	0	0·0
Do not know		2	18·2	6	50·0
Are infant present at WW (*n* 35)	30				
Yes		11	100·0	7	36·8
No		0	0·0	7	36·8
Do not know		0	0·0	5	26·3
Approximation of infant age (*n* 24)	21				
1–3 months		0	0·0	0	0·0
4–7 months		8	72·9	5	50·0
8+ months		0	0·0	0	0·0
Do not know		3	27·3	5	50·0
How are they referred to WW (*n* 25)	22				
Self-referral		2	18·2	2	18·2
GP referral		0	0·0	0	0·0
Public health nurse		5	45·5	4	36·4
Community dietitian referral		0	0·0	0	0·0
Other		4	36·4	5	45·5
Perceptions of attendees’ feedback (*n* 12)	12				
Excellent		7	63·6	0	0·0
Very good		3	27·3	1	100·0
Good		1	9·1	0	0·0
Poor		0	0·0	0	0·0
Very poor		0	0·0	0	0·0

WW, weaning workshops; SES, socio-economic status; GP, general  practitioner.

**Table 4:** Weaning workshop (WW) format, content, development and delivery of WW

		Delivered a WW (*n* 30)			
		Yes (*n* 11, 36·7 %)		No (*n* 19, 63·3 %)	
Variable	*n*	*n*	**%**	*n*	**%**
Who delivers WW (*n* 25)	21				
Community dietitian only		1	9·1	0	0·0
Public Health Nurse only		1	9·1	6	60·0
Community dietitian and public nurse		4	36·4	2	20·0
Do not know		5	45·5	2	20·0
Frequency of WW (*n* 40)	30				
Once a month		4	36·4	4	21·1
Once a fortnight		0	0·0	0	0·0
Every 4 months		2	18·2	0	0·0
Twice a year		1	9·1	0	0·0
Never		0	0·0	8	42·1
Do not know		4	36·4	7	36·8
Topics discussed at WW (*n* 24)	21				
Timing of introduction of solid foods		0	0·0	0	0·0
Responding to infant cues		0	0·0	0	0·0
Stages of food textures		2	18·2	3	30·00
Allergies		0	0·0	0	0·0
Baby-led weaning		0	0·0	1	10·0
All the above		6	54·6	0	0·0
Other		3	27·3	1	10·0
Do not know		0	0·0	5	50·0
Resources used at WW (*n* 23)	21				
Videos		0	0·0	0	0·0
Power point slides		0	0·0	0	0·0
Cooking demo		1	9·1	1	10·0
Healthy Ireland ‘Feeding your baby’ booklet		2	18·2	2	20·0
101 Square meals cookbook		0	0·0	0	0·0
Self-developed booklets/leaflets		0	0·0	0	0·0
Food display		0	0·0	0	0·0
All the above		1	9·1	0	0·0
Other		7	63·6	0	0·0
Do not know		0	0·0	7	70·0
How WW are developed (*n* 30)	30				
Self-developed		0	0·0	0	0·0
By HSE primary care dietitian in your area		3	27·3	0	0·0
By another HCP		1	9·1	2	10·5
Adapted from another HSE area		4	36·4	1	5·3
Do not know		3	27·3	16	84·2
How WW are promoted (*n* 25)	22				
Social media		0	0·0	0	0·0
Community centre		1	9·1	1	9·1
Suggested by HCP		6	54·6	6	54·6
Other		4	36·6	0	0·0
Do not know		0	0·0	4	36·6
How WW are evaluated (*n* 19)	19				
Informal participant feedback		3	27·3	0	0·0
Formal evaluation of opinions		3	27·3	0	0·0
Formal evaluation of weaning knowledge		1	9·1	0	0·0
Formal evaluation of weaning self-efficacy		1	9·1	0	0·0
Other		3	27·3	0	0·0
Do not know		0	0·0	8	100·0
Cost of WW (*n* 21)	21				
No cost		4	36·4	2	20·0
€0–50		0	0·0	0	0·0
€51–100		0	0·0	0	0·0
€101–200		2	18·2	0	0·00
€201–500		0	0·0	0	0·0
Other		2	18·2	0	0·0
Do not know		2	18·2	8	80·0
Time to travel to WW (*n* 10)	10				
0–10 min		3	3·3	1	100·0
11–20 min		0	0·0	0	0·0
21–40 min		4	44·4	0	0·0
41–60 min		2	22·2	0	0·0
> 60 min		0	0·0	0	0·0
Do not know		0	0·0	0	0·0
Time to prepare for WW (*n* 12)	12				
0–10 min		2	18·2	0	0·0
11–20 min		2	18·2	1	100·0
21–40 min		2	18·2	0	0·0
41–60 min		4	36·4	0	0·0
> 60 min		1	9·1	0	0·0
Do not know		0	0·0	0	0·0
Time to close WW (*n* 11)	11				
0–10 min		3	30·0	0	0·0
11–20 min		5	50·0	1	100·0
21–40 min		2	20·0	0	0·0
41–60 min		0	0·0	0	0·0
> 60 min		0	0·0	0	0·0
Do not know		0	0·0	0	0·0
Other time needed for WW (*n* 4)	4				
0–10 min		0	0·0	0	0·0
11–20 min		2	66·7	0	0·0
21–40 min		0	0·0	0	0·0
41–60 min		0	0·0	0	0·0
> 60 min		1	33·3	0	0·0
Do not know		0	0·0	1	100·0

HCP, health care professional: HSE, Health Service Executive.

### Dietitians’ perceptions of workshop attendees

The majority of dietitians who have delivered weaning workshops (*n* 9, 81·8 %) reported that parents who attend weaning workshops are mostly of medium SES. Of those who had delivered weaning workshops, the majority indicated that only mothers (*n* 9, 81·8 %) with their infants (*n* 11, 100·0 %), aged 4–7 months (*n* 8, 72·9 %) attended weaning workshops. Most dietitians who had not delivered a weaning workshop answered ‘I don’t know’ to most of the questions including ‘who attends weaning workshop’s (*n* 7, 53·9 %), ‘parents’ SES’ (*n* 6, 50·0 %), ‘how many attended a weaning workshop class’ (*n* 5, 45·5 %) and ‘how parents were referred’ to a weaning workshop (*n* 5, 45·5 %). Finally, the majority of dietitians who had delivered weaning workshops reported attendees’ feedback as ‘excellent’ (*n* 7, 63·6 %) (Table 3).

### Weaning workshop format, content, development and delivery of weaning workshops

Of the eleven dietitians who had previously delivered a workshop, 36·4 % (*n* 4) reported that workshops were delivered by community dieticians in collaboration with public health nurses, while 45·5 % did not know what other healthcare professionals were also involved in delivering weaning workshops (Table 4). Of dietitians with experience in delivering weaning workshops, 54·6 % reported that workshop content included discussion on the timing of introduction of solid foods; responding to infant cues; stages of food textures, allergies and baby-led weaning (*n* 6). According to the dietitians with experience, weaning workshops were evaluated using either informal participant feedback (*n* 3, 27·3 %) or formal evaluation of opinions (*n* 3, 27·3 %). For those who had delivered weaning workshops, the majority (*n* 4, 44·4 %) mostly indicated that the time taken to travel to workshops was approximately 21–40 min, and to prepare for the workshops was 41–60 min. Most dietitians who had not delivered a weaning workshop did not know how weaning workshops were developed (*n* 16, 84·2 %) or promoted (*n* 4, 36·6 %), with some indicating that promotion was through other healthcare professionals (*n* 6, 54·6 %). When asked about the cost of weaning workshops in terms of resources needed such as room hire or equipment, of those who had delivered weaning workshops, 36·4 % reported no cost (*n* 4), with 18·2 % reporting €101–€200 (*n* 2).

### Factors influencing the development and delivery of workshops

Participants identified limited resources and a lack of staffing as primary reasons that workshops were not currently offered within their area. It was highlighted that ‘*not all health centers have weaning workshops due to staff constraints’* and that some public health nurse did ‘*not have enough clinical time to run group’*. Furthermore, poor parent attendance at previous workshops was emphasised as a reason why classes had stopped *‘…poor attendance in some areas in the past hence workshops were discontinued…. Mothers and fathers were invited to attend an informal gathering to learn more about weaning, unfortunately the attendance was not always good’*

Participants also identified several factors they felt should be considered in the future development and delivery of weaning workshops. These include workshop characteristics such as appropriate content, timing, venue and mode of delivery, as well as organisational characteristics such as the availability of resources, multidisciplinary team involvement and attendee characteristics. One participant felt that weaning workshops were not needed within primary care. This data is further described in Table 5 which also provides the numbers of participants who identified each factor and sample quotes.

**Table 5:** Factors to consider in the development and delivery of workshops

Category	Factors *(n* = *number of respondents who identified this)*	Example quotes
Barriers to the implementation of workshops	*Staff constraints:* • Lack of time (*n* 5) • No available dietitians (*n* 2) • Lack of staff direction (*n* 2) *Resources:* • Funding (*n* 1) *Participants:* • Poor attendance *(n 2)*	• *‘These are not been facilitated due to inadequate staffing levels.’* • *‘Primary care team does not have the capacity to deliver weaning workshops’* • *‘None presently due to resource constraints but are currently planning to commence in one target area’* • *‘We have no funding. they are run very occasionally, maybe once a year in our area.’* • *‘I am only in post since Sept 18 and this is a half time position so presently I do not have the capacity to do weaning workshops. However, I feel it would be something that would be beneficial for the area.’* • *‘Mothers and fathers are invited to attend an informal gathering to learn more about weaning especially if they are first time mum. Unfortunately, the attendance is not always good’*
Workshop characteristics	*Content:* • Practical and interactive (*n* 5) • Standardised/structured (*n* 4) • Evidence-based (*n* 3) • Responsive feeding/industry influence/stages of textures (*n* 3)	• *’Any program should be interesting and interactive’* • *’Using existing materials to avoid duplication & save time, standardisation of material if possible’* • *’Nationally standardised weaning workshops available online and offline should be available for new parents and carers’* • *’Very important that there is more out there for parents/families re weaning advice - especially evidence-based and safe advice!’* • *’We also need to ensure messages around responsive feeding are shared with parents’* • *’Nationally agreed referral pathway with a structured programme incorporated as part of the dietetic service to primary care’* • *’Most important component re weaning is to make sure textures are introduced in a timely fashion. We see many fussy eaters who took pureed foods for far too long’*
	*Timing:* • Appropriate to child age/stage (*n* 5)	• *’To have them running a few times a year, to ensure you are able to see families at appropriate times’* • *’National weaning program - offered to all parents when their baby is between 3 and 6 months’*
	*Venue:* • Appropriate baby-friendly location (*n* 7)	• *’Because Mums attend with children it is necessary to have the workshop in a room that caters for children’*
	*Mode of delivery:* • Accessible and free (*n* 4) • Use of peer leaders (*n* 1)	• *’That they are accessible to all and free of charge’* • *’I do enjoy delivering the workshops myself, but appreciate that to scale up the trained [peer] tutor led approach would work best’*
Resources	• Staff time/availability (*n* 8) • Staff training/resources (*n* 3) • Administration support (*n* 2)	• *’Dedicated committed staff so it won’t flounder…a few key people driving it’* • *’Staffing such as releasing staff to deliver and continue to invest and develop the programme…allow staff to gain skills such as facilitation’* • *’Adequate funding of the project is essential to its continued success’*
Multidisciplinary team (MDT) involvement	• MDT involvement to facilitate recruitment/referral (*n* 7) • MDT involvement in development/delivery (*n* 5)	• *’It needs to be inclusive of all relevant disciplines not just Public Health Nursing driven and led’* • *’Joint delivery between PHN and dietitian works well - has strengthened the relationship between nursing and dietetics’* • *’Could be alternated between PHN, peer leader and Dietitian, would need PHN buy in’*
Participant characteristics	• Engaging participants from low SES (*n* 6) • Participant attendance (*n* 2)	• *’How to engage those most in need of the information. Over the past 19 years it is middle-class well-informed parents that attend these workshops’* • *’Access for lower socio-economic groups. They are under such social stress that they don’t take up groups/workshops.’* • *’The mums that attend is generally very interested in good nutrition and probably have read widely on the subject prior to attending. Unfortunately, it is difficult to get the mums who really need education on baby weaning to attend’*

Overall, many participants regardless of their experience of delivery highlighted that workshop should be standardised and structured, and that content and associated materials should be practical and interactive, evidence-based and cover topics like responsive feeding, the potential influence of the infant feeding industry and stages of textures. For example, one participant wrote that there is a need for a *’nationally agreed referral pathway with a structured programme incorporated as part of the dietetic service to primary care’.* Dietitians also identified that workshops should be appropriate to the child age/stage, e.g. to be run a few times a year to engage with parents and caregivers at appropriate times and be held in child-friendly venues, at no cost to participants with one stating ‘*because Mums attend with children it is necessary to have the workshop in a room that caters for children’*. Regarding the resources needed to develop and implement these workshops, participants identified that the availability of staff including administrative support was crucial. They also stated that multidisciplinary team involvement was crucial both in terms of recruiting or referring attendees to the workshop, and in the development and delivery of workshops, as one pa’ticipant remarked, *’it needs to be inclusive of all relevant disciplines’’.* Finally, in relation to attendee characteristics, dietitians stated the importance of running workshops for parents and/or caregivers with the greatest need, e.g. with a particular focus on engaging families from lower SES backgrounds, *’The mums that attend is [sic] generally very interested in good nutrition and probably have read widely on the subject prior to attending. Unfortunately, it is difficult to get the mums who really need education on baby weaning to attend’.*

## Discussion

This study provides an overview of current practice regarding the delivery of weaning workshops within the Irish primary healthcare service. The study also provides insight into the experiences and opinions of primary care community dietitians regarding the development and delivery of weaning workshops in terms of optimal content and delivery options. Overall, only eleven dietitians reported experience of personally delivering weaning workshops. Sixteen dietitians reported that workshops were run in their area with variable frequency, with ten reporting that workshops were never run in their area. Participants reported that mostly only mothers of medium SES attended weaning workshops when infants were aged between 4 and 7 months, and that feedback from attendees was mostly positive.

The results provide insight into barriers faced by service areas while identifying key factors that need to be considered in the future development and delivery of weaning workshops. Dietitians identified staff constraints, limited resources and poor participant attendance as reasons to why weaning workshops are not held or have been discontinued. Previous research identified similar barriers such as the time required to be involved in advocacy outside of clinical practice, a lack of knowledge on how to connect to their communities and a lack of support from their workplaces^(37)^. Public health nurses and dietitians need support from their health care institutions in the form of education, time and funding to facilitate their health promotion efforts. Furthermore, to increase community engagement suitable accessible locations need to be used along with important promotion. Furthermore, a coordinated list of community resources need to be compiled so that healthcare professionals can refer patients to infant feeding/complementary feeding education workshops across service areas^(38)^. While dietitians in this study identified factors that should be considered in the future development and delivery of weaning workshops such as content, timing and venue, the availability of resources, multidisciplinary involvement and attendee characteristics, future research needs to address the broader implementation requirements for sustained long-term implementation^(39)^.

This study highlights substantial variability in current practice regarding weaning workshops in Irish primary care, with workshops being run in some areas by dietitians but not in others, as well as a lack of standardisation regarding the provider, content and frequency of workshops where workshops are being delivered. We also identified a distinct lack of clarity and knowledge amongst dietitians regarding what current practice is regarding weaning workshops, with many reporting ‘don’t know’ responses. Future research needs to review infant feeding recommendations to create evidence-based education with specific learning outcomes for participants, creating a programme of education which is consistent across service areas. Furthermore, moving educational roles from healthcare professionals to lay educators/peer leaders could create more sustainable implementation, helping to overcome time constraints faced by healthcare professionals. However, different funding approaches would be required to train lay educators and to provide the necessary resources to implement and sustain weaning workshops within service areas.

Like other studies in this area, participants agreed on several key factors to be considered for the future development and implementation of weaning workshops such as the timing of weaning workshops and ensuring appropriateness of the workshop to the child age/stage was a crucial factor identified by dietitians in this study. Previous research has indicated that mothers are more responsive to personalised approaches within infant feeding interventions^(40,41)^ and that tailoring workshops to target different stages of infant development is important to optimise their effectiveness^(29)^. Previous intervention studies have indicated that interventions initiated antenatally^(42)^ or up to 10 weeks after birth^(43,44)^ can be effective in promoting appropriate infant feeding^(29)^. However, this study found that workshops currently offered involve infants between 4–7 months of age, with no dietitian reporting workshops delivered to parents with infants aged 0–3 months. Given the fact that infants are often introduced to solids before 4 months, and sometimes as early as 12 weeks as outlined earlier, this further highlights the importance of carefully considering the timing of such programmes^(3,4,6,7)^. Future research on effective timing and frequency of intervention dose is required to ensure that all stages of development are targeted to improve infant feeding practices and nutritional habits.

This study also highlights the importance of targeting weaning workshops and infant feeding programmes towards the areas of most need. A common theme identified by participants was the need to engage parents and caregivers from lower SES backgrounds; however, dietitians also reported that workshops are currently predominantly attended by mothers of medium SES. Existing guidance emphasises the importance of different intervention approaches which can focus on deprived groups^(29)^ to target the recurring relationship that exists between SES and inappropriate infant feeding practices^(35,45,46)^. Previous studies have identified maternal education and SES as factors which predict poor infant feeing behaviours^(47,48)^. Given that participants identified the availability of resources (e.g. staff time, cost) as a key factor influencing the development and delivery of weaning workshops in resource-constrained settings, it is likely to be particularly important to prioritise the provision of workshops for parents and caregivers from more deprived backgrounds. A previous evaluation of infant feeding workshops amongst women living in areas of high deprivation found that a relatively short education intervention can enhance women’s knowledge and understanding of weaning^(29)^. While demographic factors are unmodifiable, there is scope to focus on and influence modifiable factors such as women’s beliefs about infant feeding^(35,45)^. Therefore, the future development and implementation of weaning workshops should consider important aspects such as accessibility and cost, providing workshops in appropriate locations at different times, free of charge that does not incur additional expenses on attendees (e.g. childcare, parking), with more concerted efforts to specifically engage with and target families of lower SES.

Dietitians in this study perceived mothers’ feedback on these weaning workshops as ‘excellent’. Similarly, a study that evaluated infant feeding workshops for women from deprived areas found the workshops were generally rated positively while also being seen as an accessible source of education^(29)^. Furthermore, several other studies found that after attending educational and practical workshops on infant feeding and complementary feeding women appeared to have improved knowledge, understanding and confidence, with many following the current infant feeding recommendations^(40,41,44)^. While data such as this is encouraging, future research should formally evaluate attendees’ experiences, levels of knowledge and infant feeding behaviours prior to and following attendance at weaning workshops to ensure that content is clear and comprehensive.

### Limitations

A main limitation of this survey is that information provided on parent/primary caregiver attendees is from dietitian self-report and as we did not collect data on time since delivering workshops, it may also be influenced by participant recall. We did not have the resources to also collect data from parent/caregivers, therefore future research should explore weaning workshops with attendees to obtain a more comprehensive understanding of the phenomenon in question. In items that allowed for multiple responses (‘tick all that apply’), variables were re-categorised for the analysis, easy interpretation and presentation of results. However, by doing this, some information is lost, so caution must be used when interpreting the results. ‘Tick all that apply’ answer format in questionnaires do not require respondents to differentiate between the answer responses, therefore a series of forced choice Yes/No questions should be used as recommended in future research^(49)^. In addition, only eleven participants reported first-hand experience of delivering weaning workshops and as such, some responses may have been more directly informed by tacit experience of delivering weaning workshops than others. However, our chi-squared tests showed no observed patterns of difference between the overall responses of those with and without experience, and we felt that it is important to include the views of all participants, as some may have been involved in commissioning or organising rather than delivery or may have drawn on other relevant experience. We also felt it was important to include the views of all dietitians who might potentially be involved in the delivery of future workshops to give a more inclusive overview of potential factors and barriers/facilitators to consider.

## Conclusions

This study provides a snapshot of current delivery of community-based weaning workshops within the Irish health service. Using a cross-sectional survey, community-based dietitians perceived participants’ feedback on weaning workshops as excellent, while highlighting issues around optimal timing and delivery of workshops. Furthermore, the findings highlight that future research needs to focus on how to engage families of lower SES and the impact of these workshops on behaviour change, taking into consideration key factors such as accessibility and cost. Although based within an Irish setting, the need to support complementary feeding and subsequent rationale for establishing weaning workshops are extremely pertinent internationally. Given the dearth of evidence in this area, the insights provided by participants of this study regarding the development and delivery of weaning workshops, in terms of optimal content and delivery options, will make a valuable contribution to this field.

## Supplementary material

For supplementary material accompanying this paper visit https://doi.org/10.1017/S1368980022000477. click here to view supplementary material

## References

[1] WHO (2003) Global Strategy for Infant and Young Child Feeding. Geneva: World Health Organization.

[2] World Health Organization (2003) Complementary Feeding: Report of the Global Consultation, and Summary of Guiding Principles for Complementary Feeding of the Breastfed Child. Geneva: World Health Organization.

[3] Spaniol AM , da Costa THM , de Moura Souza A et al. (2021) Early consumption of ultra-processed foods among children under 2 years old in Brazil. Public Health Nutr 24, 3341–3351. 33222721 10.1017/S1368980020004759 PMC10195384

[4] Castro PD , Kearney J & Layte R (2015) A study of early complementary feeding determinants in the Republic of Ireland based on a cross-sectional analysis of the Growing Up in Ireland infant cohort. Public Health Nutr 18, 292–302. 24642376 10.1017/S1368980014000329 PMC10271045

[5] Tarrant RC , Younger KM , Sheridan-Pereira M et al. (2010) Factors associated with weaning practices in term infants: a prospective observational study in Ireland. Br J Nutr 104, 1544–1554. 20598218 10.1017/S0007114510002412

[6] McSweeney M & Kevany J (1982) A national survey of infant feeding practices in Ireland 1981: a preliminary report. Irish Med J 75, 452–455. 7161023

[7] Tarrant R (2008) An Investigation of the Diets of Infants Born in Ireland During the First Six Months of Life. Dublin: Dublin Institute of Technology.

[8] Fall CH , Borja JB , Osmond C et al. (2011) Infant-feeding patterns and cardiovascular risk factors in young adulthood: data from five cohorts in low- and middle-income countries. Int J Epidemiol 40, 47–62. 20852257 10.1093/ije/dyq155 PMC3043278

[9] Gutiérrez-Camacho C , Méndez-Sánchez L , Klünder-Klünder M et al. (2019) Association between sociodemographic factors and dietary patterns in children under 24 months of age: a systematic review. Nutrients 11, 2006. 31454895 10.3390/nu11092006 PMC6770717

[10] Matvienko-Sikar K , Kelly C , Sinnott C et al. (2018) Parental experiences and perceptions of infant complementary feeding: a qualitative evidence synthesis. Obes Rev 19, 501–517. 29243375 10.1111/obr.12653

[11] Spyreli E , McKinley MC & Dean M (2021) Parental considerations during complementary feeding in higher income countries: a systematic review of qualitative evidence. Public Health Nutr 24(10) 2834–2847. 33877043 10.1017/S1368980021001749 PMC9884778

[12] Waters E , de Silva-Sanigorski A , Burford BJ et al. (2011) Interventions for preventing obesity in children. Cochrane Database Syst Rev 12, CD001871. 10.1002/14651858.CD001871.pub3 22161367

[13] Redsell SA , Edmonds B , Swift JA et al. (2016) Systematic review of randomised controlled trials of interventions that aim to reduce the risk, either directly or indirectly, of overweight and obesity in infancy and early childhood. Matern Child Nutr 12, 24–38. 25894857 10.1111/mcn.12184 PMC5029770

[14] Graziose MM , Downs SM , O’Brien Q et al. (2018) Systematic review of the design, implementation and effectiveness of mass media and nutrition education interventions for infant and young child feeding. Public Health Nutr 21, 273–287. 29081315 10.1017/S1368980017002786 PMC10260785

[15] Toomey E , Matvienko-Sikar K , Heary C et al. (2019) Intervention fidelity within trials of infant feeding behavioral interventions to prevent childhood obesity: a systematic review. Ann Behav Medicine 53, 75–97. 10.1093/abm/kay021 29796664

[16] HSE (2016) National Healthy Childhood Programme, Newsletter. http://hdlhandlenet/10147/620988 (accessed February 2022).

[17] HSE (2016) The Nurture Programme – Infant Health and Wellbeing. https://wwwhseie/eng/health/child/nurture/abouthtml (accessed February 2022).

[18] World Health Organization (2018) Primary Health Care: Closing the Gap between Public Health and Primary Care through Integration. Geneva: World Health Organization.

[19] World Health Organization (2014) Global Status Report on Noncommunicable Diseases. Geneva: World Health Organization.

[20] Vine M , Hargreaves MB , Briefel RR et al. (2013) Expanding the role of primary care in the prevention and treatment of childhood obesity: a review of clinic- and community-based recommendations and interventions. J Obes 2013, 172035. 23710345 10.1155/2013/172035 PMC3655557

[21] Toomey E , Flannery C , Matvienko-Sikar K et al. (2021) Exploring healthcare professionals’ views of the acceptability of delivering interventions to promote healthy infant feeding practices within primary care: a qualitative interview study. Public Health Nutr 24, 2889–2899. 33317663 10.1017/S1368980020004954 PMC9884767

[22] Furlong M & McGilloway S (2012) The Incredible Years Parenting program in Ireland: a qualitative analysis of the experience of disadvantaged parents. Clin Child Psychol Psychiatry 17, 616–630. 22104366 10.1177/1359104511426406

[23] Gardner F , Leijten P , Mann J et al. (2017) Could scale-up of parenting programmes improve child disruptive behaviour and reduce social inequalities? Using individual participant data meta-analysis to establish for whom programmes are effective and cost-effective. Public Health Res 5(10). 29227603

[24] Leijten P , Raaijmakers MAJ , Orobio de Castro B et al. (2017) Effectiveness of the Incredible Years Parenting program for families with socioeconomically disadvantaged and ethnic minority backgrounds. J Clin Child Adolesc Psychol 46, 59–73. 25985392 10.1080/15374416.2015.1038823

[25] Reid MJ , Webster-Stratton C & Baydar N (2004) Halting the development of conduct problems in Head Start children: the effects of parent training. J Clin Child Adolesc Psychology 33, 279–291. 10.1207/s15374424jccp3302_10 15136193

[26] Love P , Laws R , Litterbach E et al. (2018) Factors influencing parental engagement in an early childhood obesity prevention program implemented at scale: the infant program. Nutrients 10, 509. 29671815 10.3390/nu10040509 PMC5946294

[27] Gardner F , Burton J & Klimes I (2006) Randomised controlled trial of a parenting intervention in the voluntary sector for reducing child conduct problems: outcomes and mechanisms of change. J Child Psychol Psychiatr 47, 1123–1132. 10.1111/j.1469-7610.2006.01668.x 17076751

[28] Gardner F , Hutchings J , Bywater T et al. (2010) Who benefits and how does it work? Moderators and mediators of outcome in an effectiveness trial of a parenting intervention. J Clin Child Adolesc Psychol 39, 568–580. 20589567 10.1080/15374416.2010.486315

[29] Andrews E , Symon A & Anderson A (2015) ‘I didn’t know why you had to wait’: an evaluation of NHS infant-feeding workshops amongst women living in areas of high deprivation. J Hum Nutr Dietetics 28, 558–567. 10.1111/jhn.12269 25208593

[30] Hesketh KD , Salmon J , McNaughton SA et al. (2020) Long-term outcomes (2 and 3.5 years post-intervention) of the INFANT early childhood intervention to improve health behaviors and reduce obesity: cluster randomised controlled trial follow-up. Int J Behav Nutr Phys Act 17, 1–10. 32711523 10.1186/s12966-020-00994-9 PMC7382091

[31] Blake-Lamb TL , Locks LM , Perkins ME et al. (2016) Interventions for childhood obesity in the first 1000 d a systematic review. Am J Prev Med 50, 780–789. 26916260 10.1016/j.amepre.2015.11.010 PMC5207495

[32] Frongillo EA (2017) Evaluation of programs to improve complementary feeding in infants and young children. Matern Child Nutr 13, e12436. 29032620 10.1111/mcn.12436 PMC6865996

[33] Martin SL , McCann JK , Gascoigne E et al. (2020) Mixed-methods systematic review of behavioral interventions in low-and middle-income countries to increase family support for maternal, infant, and young child nutrition during the first 1000 d. Curr Dev Nutr 4, nzaa085. 32607463 10.1093/cdn/nzaa085 PMC7311193

[34] Von Elm E , Altman DG , Egger M et al. (2014) The Strengthening the Reporting of Observational Studies in Epidemiology (STROBE) Statement: guidelines for reporting observational studies. Int J Surg 12, 1495–1499. 25046131 10.1016/j.ijsu.2014.07.013

[35] Alder EM , Williams FL , Anderson AS et al. (2004) What influences the timing of the introduction of solid food to infants? Br J Nutr 92, 527–531. 15469658 10.1079/bjn20041212

[36] Hsieh H-F & Shannon SE (2005) Three approaches to qualitative content analysis. Qual na research 15, 1277–1288. 10.1177/1049732305276687 16204405

[37] McPherson ME , Mirkin R , Heatherley PN et al. (2012) Educating health care professionals in advocacy for childhood obesity prevention in their communities: integrating public health and primary care in the Be Our Voice project. Am J Public Health 102, e37–e43. 10.2105/AJPH.2012.300833 PMC3464860 22698054

[38] Boyle M , Lawrence S , Schwarte L et al. (2009) Health care providers’ perceived role in changing environments to promote healthy eating and physical activity: baseline findings from health care providers participating in the healthy eating, active communities program. Pediatrics 123, S293–S300. 19470606 10.1542/peds.2008-2780H

[39] Toomey E , Matvienko-Sikar K , Doherty E et al. (2020) A collaborative approach to developing sustainable behaviour change interventions for childhood obesity prevention: development of the Choosing Healthy Eating for Infant Health (CHErIsH) intervention and implementation strategy. Br J Health Psychol 25, 275–304. 31999887 10.1111/bjhp.12407

[40] Black MM , Siegel EH , Abel Y et al. (2001) Home and videotape intervention delays early complementary feeding among adolescent mothers. Pediatrics 107, e67. 11331717 10.1542/peds.107.5.e67

[41] Brophy-Herb HE , Silk K , Horodynski MA et al. (2009) Key theoretical frameworks for intervention: understanding and promoting behavior change in parent–infant feeding choices in a low-income population. J Prim Prev 30, 191–208. 19283484 10.1007/s10935-009-0169-9

[42] McCormick F , Moreton J , D’Souza L et al. (2007) The Effectiveness of Public Health Interventions to Improve the Nutrition of Young Children Aged 6–24 Months. London: NICE.

[43] Hoare K , Wright C , Wilson P et al. (2002) Disseminating weaning messages: an intervention trial. Br J Community Nurs 7, 196–200. 11979198 10.12968/bjcn.2002.7.4.10225

[44] Watt R , Tull K , Hardy R et al. (2009) Effectiveness of a social support intervention on infant feeding practices: randomised controlled trial. J Epidemiol Community Health 63, 156–162. 19141661 10.1136/jech.2008.077115

[45] Bolling K , Grant C , Hamlyn B et al. (2007) Infant Feeding Survey 2005. London: The Information Centre for Health and Social Care.

[46] Excellence NIfC (2008) Public Health Guidance 11: Improving the Nutrition of Pregnant and Breastfeeding Mothers and Children in Low-Income Households. London: NICE.

[47] Wright CM , Parkinson K & Drewett R (2004) Why are babies weaned early? Data from a prospective population based cohort study. Arch Dis Childhood 89, 813–816. 15321854 10.1136/adc.2003.038448 PMC1763205

[48] Wijndaele K , Lakshman R , Landsbaugh JR et al. (2009) Determinants of early weaning and use of unmodified cow’s milk in infants: a systematic review. J Am Dietetic Assoc 109, 2017–2028. 10.1016/j.jada.2009.09.003 19942019

[49] Dillman DA , Smyth JD , Christian LM et al. (2003) Multiple Answer Questions in Self-Administered Surveys: The Use of Check-All-That-Apply, Forced-Choice Question Formats. San Francisco, CA: American Statistical Association.

